# The Issue of Unconscious Bias in Referee Decisions in the National Rugby League

**DOI:** 10.3389/fspor.2021.739570

**Published:** 2021-09-30

**Authors:** Katherine A. O'Brien, John Mangan

**Affiliations:** ^1^School of Exercise and Nutrition Sciences, Queensland University of Technology, Brisbane, QLD, Australia; ^2^Faculty of Business, Economics and Law, The University of Queensland, Brisbane, QLD, Australia

**Keywords:** referee bias, rugby league, home advantage, educational processes, learning

## Abstract

Referees are appointed to be impartial sporting figures. They are trained to provide leadership and guidance, interpret infractions, adjudicate rules, all while maintaining the highest levels of objectivity and sense. However, when decisions are driven by individual heuristics, limited information, context, goal motivations, emotions, time pressures and cognitive load, it can be difficult to discern how and why particular referee judgements are made. In this paper, we draw on data from the major Rugby League competition in Australia between the years 1978 to 2019 to examine whether clubs fare significantly better or worse under particular National Rugby League referees. We examine potential causes that might contribute to the referee effect and ask why, after years of specialist training and game assessments, are rugby league referees, even at the elite professional level, still processing information in preferential ways.

## Introduction

Referees are appointed to be impartial sporting figures. They are trained to provide leadership and guidance, interpret infractions, adjudicate rules, all while maintaining the highest levels of objectivity and sense (Lirgg et al., 2016; Hancock et al., 2018). Their ability to prioritize and process information, at the right time, in order to select the appropriate response from competing task demands is a sign of their dependability and a reflection of their perceptual-cognitive expertise (Moore et al., 2019). However, when decisions are driven by individual heuristics, limited information, context, goal motivations, emotions, time pressures and cognitive load (Hill and Windmann, 2014; Osório, 2020), it can be difficult to discern how and why particular referee judgements are made.

Four important streams of work currently characterize the presence of judgement and decision making in sports (Raab et al., 2019). Influenced by different disciplines such as psychology and neuroscience, any one of these avenues (i.e., economic, social judgement, ecological and cognitive approaches to decision making) could characterize how and why particular referee decisions are made. For example, information gaps have been found to cause incongruity and ambiguity which influences how performers, such as referees, perceive events (Agarwal, 2020). Decision-making is subject to social influences and performers own unique interpretations, attitudes, hidden biases, predictions and demonstrated preferences (Thuraisingham, 2013; Rhodes et al., 2017; Agarwal, 2020). Paradoxically, even illusionary memories have been found to impact decision-making during game play (Hill and Windmann, 2014). Recent research has also reported that biases or shortcut strategies used to manage complex information can arise as a result of performers trying to quickly identify and process fast-paced visual, verbal, and behavioral cues (Moore et al., 2019; Albanese et al., 2020; Osório, 2020). Because sport referees must make judgments and decisions under time constraints, in complex environments and often under ambiguous circumstances, the inclination or prejudice for or against one person or sporting group would appear to be guaranteed (Erikstad and Johansen, 2020).

Systematic decisions in favor of certain players or sporting groups, which can result from conscious bias as well as from human error or incompetence, may have significant economic impacts on sport organizations in areas such as ticket sales, prize money, merchandise, broadcast revenue and recruitment (Agarwal, 2020; Albanese et al., 2020). Examining the potentially tapered focus of professional sport referees also highlights that these performers receive material incentives such as match payments and appointments stemming from match reviews. Given the somewhat opaque and potentially subjective nature of the system for evaluating and compensating referees (Price et al., 2012), it is difficult to say, without further research, what the precise cost and benefits of biases such as unconscious bias are for referees and the organization itself. Indeed, it should be considered that sport governing bodies might even reward unconscious bias, or at least only provide a weak disincentive for bias related to areas such as home advantage, if bias substantially benefits the business in terms of increased ticket sales, club memberships, and broadcast rights (Price et al., 2012; Areni, 2014). Answering whether professional referees make biased decisions and understanding the causes that lead to professional referees digressing from their role as impartial sporting arbiters is therefore important (Dohmen and Sauermann, 2016).

In this paper, we focus on referees operating in the sport of rugby league. We define what we mean by unconscious bias and highlight how a growing body of literature has examined how unintentional predispositions are shaping decision making in sports, before singling out home advantage as the most likely indicator of unconscious bias. Section 2 examines data on home advantage in the National Rugby League (NRL) competition in Australia and shows that even among elite professional referees, home advantage differentials vary widely around the average or expected values. While differences such as these do not necessarily imply bias, the empirical model developed in Section 3, using a series of control variables to decompose the “referee effect” from other factors that contribute to the home advantage points spread, illustrates that while some penalties are obvious, many are the result of referee interpretation. Section 4 considers potential causes that contribute to the referee effect and examines the perverse incentives and moral hazards present in the current system of referee development and asks why, after years of specialist training and game assessments, are rugby league referees, even at the elite professional level, still processing information in preferential ways.

## Section 1—Unconscious Bias in Sport Refereeing

### What Is Implicit Bias?

Implicit biases are learned stereotypes that are automatic, unintentional, deeply engrained, and able to influence behavior (Fiarman, 2016; Backhus et al., 2019). They refer to individuals' lack of awareness of the effects of their own actions on other people or social institutions (Beugr, 2018). Unlike explicit biases, where people are aware of their prejudices and attitudes toward certain groups or events, implicit biases occur below the level of consciousness. Their signature feature is that they represent automatic and unconscious cognitive processes; they are not direct, deliberate, controlled, or intentional self-assessments (Nosek et al., 2011; Agarwal, 2020). They also illustrate how people's reports of the causes of their behavior can be stated confidently and incorrectly simultaneously (Nosek et al., 2011). Implicit biases, conversationally referred to as unconscious biases, are therefore problematic to capture and accurately pinpoint because they are hidden and can often be in complete contrast to what people consider their beliefs and associations to be (Agarwal, 2020).

### What Is Bias in Professional Refereeing/What Do We Already Know?

Unconscious bias in the context of professional refereeing may mean that learned stereotypes, deeply ingrained within their beliefs, influence the way in which individual referees automatically engage with players and situations. From this viewpoint, judgement biases could be described as the subconscious overweighting of some aspects of information and underweighting or neglect of others (Morewedge and Kahneman, 2010), relative to rules and situations. For example, referees have been shown to be persuaded by crowd reactions (Page and Page, 2010; Erikstad and Johansen, 2020). Racial bias has been found among National Basketball Association referees (Price and Wolfers, 2010), Major League Baseball umpires (Parsons et al., 2011), and National Collegiate Women's Basketball referees (Dix, 2019). Baseball umpires also display how high-status players were rewarded with correct decisions even when their performances were undeserving (Kim and King, 2014). Similarly, Findlay and Ste-Marie (2004) found that rankings used to determine a Canadian skaters final placement were better when skaters were evaluated by judges who knew of the skaters positive reputation. Researchers have also extensively analyzed other determinants of referee bias such as social pressure from the crowd and media (Myers, 2014; Webb, 2018), cultural closeness of the referee to the team (Pope and Pope, 2015; Nezlek et al., 2019), players' height (Gift and Rodenberg, 2014), and uniform color (Dijkstra et al., 2018). Although the external validity of the results from these studies on bias remain an open question (Osório, 2020), they are at least suggestive that unconscious biases may play a pivotal role in shaping referees' evaluations of others, particularly when part of split-second, high-pressure sporting plays.

### Singling Out Home Advantage

A large proportion of studies on referee bias have focused on home team advantage (Burnett et al., 2017; Albanese et al., 2020). The phenomenon by which there is an apparent advantage conferred to the home team by referees is broadly accepted and well documented for a wide variety of sports (Ribeiro et al., 2016). Three major determinants have been postulated to cause the home advantage effect: crowds (e.g., crowd partisanship, crowd noise), travel fatigue and familiarity with the stadium (Pollard and Pollard, 2005). Of these, situational influences related to crowd effects such as density and home crowd noise have been substantially reviewed (Page and Page, 2010; Myers, 2014; Dosseville et al., 2016; Erikstad and Johansen, 2020). When taken together, these studies suggest that referees might be biased by crowd reaction, favoring the home team, and thereby contributing to home advantage (Dosseville et al., 2016). In essence, the intuitive or subconscious overweighting of some aspects of information such as the strict enforcement of laws or crowd noise and underweighting or neglect of other aspects to ensure the flow of the game might simply reflect a referee's unconscious bias.

## Section 2—The Empirics of Unconscious Bias in Rugby League

This section draws on data from the major Rugby League competition in Australia between the years 1978 to 2019. That period saw the development of Rugby League in Australia from State and City based competitions to a quasi-National and International competition under the auspices of the National Rugby League (NRL). During this period, over 70 persons have acted as elite professional referees ranging from a select group (*n* = 5) who have refereed over 350 games to a group of 30 with <50 “A Grade/NRL” professional games.

There are a number of commonly held beliefs in professional Rugby League that have almost reached axiomatic status such as “forwards win big matches”; ‘a team cannot win a premiership without a strong spine” and “you need to lose a premiership to win one” but, probably the most enduring axiom is “home team advantage.” As outlined earlier, the potential sources of home team advantage are several including greater crowd support, reduction in travel time, crowd density and familiarity of surroundings. The role of the referee in the perpetuation of home advantage, however, is open to debate. For example, Boyko et al. (2007) found evidence that referees on average do tend, in an implicit sense, to enhance home team advantage; that is home teams tend to score more points than their expected value and to surrender less than their expected value of points against. Similarly, Unkelbach and Memmert (2010) demonstrated the possible contribution of crowd noise to the home advantage via soccer referee's decisions. Both of these studies, of course, depend on the generalized existence and quantification of home advantage.

A number of studies have attempted to measure the extent of home advantage within the NRL. McGuckin et al. (2015) for example defined home advantage as teams winning more that 50% of home games. However, there are a number of limitations to this type of definition. In particular, home advantage does not necessarily equate with winning. Poorly performing teams who lose but score more points or have less points scored against them at home are still beneficiaries of home advantage even though this does not translate into wins. Certainly, if home advantage is important, you would expect teams to have a higher likelihood of winning at home^1^ but a better indicator of home advantage, which allows for the possibility of poorly performing teams still enjoying some form of home advantage would appear to be in the points scored and conceded against these teams in away games. On that basis we could define home advantage in attack as:


(1) $$\begin{array}{l} \left(\overset{n}{\underset{i}{\sum }}PSH_{i}-\overset{n}{\underset{i}{\sum }}PSA_{i}\right)/\overset{n}{\underset{i}{\sum }}PSH_{i} \end{array}$$

Where $\overset{n}{\underset{i}{\sum }}PSH_{i}$ = the sum of points scored at home by that team over n games and $\overset{n}{\underset{i}{\sum }}PSA_{i}=$ the sum of the points scored by that team in away games.


(2) $$\begin{array}{l} \text{Similarly},\text{ home advantage in defense}=(\sum_{i}^{n}PSA_{i}-\\ \;\sum_{i}^{n}PSH_{i})/\sum_{i}^{n}PSA_{i} \end{array}$$

Using these formulas, the existence of home advantage in both attack and defense is shown in Table 1.

**Table 1:** Average value of home advantage in attack and defense in the NRL.

**Team**	**Home advantage in attack %**	**Home advantage in defense %**
Melbourne Storm	21	34
Brisbane Broncos	24	11
Canberra Raiders	25	18
Manly Warringah Sea Eagles	22	17
Wests Tigers	9	19
Canterbury Bankstown Bulldogs	9	12
Auckland Warriors	12	26
Sydney Roosters	9	21
Parramatta Eels	22	21
Penrith Panthers	17	18
St George Illawarra Dragons	16	16
Newcastle Knights	21	34
Cronulla Sutherland Sharks	15	29
North Queensland Cowboys	15	29
Gold Coast Titans	11	16
South Sydney Rabbitohs	13	13
Balmain Tigers	20	24

*Data derived from the University of Queensland NRL database (2020)*.

Table 1 examines the percentage difference between points scored by a team at home and points scored away and the percentage difference between points conceded at home and points conceded away. For every team there appears to be an advantage playing at home. All teams reported more points scored and less points conceded when playing at home. It might be thought that the more successful teams in terms of winning percentage would enjoy the largest home advantage, but, on first inspection of the data in Table 1, this does not appear to be the case. In terms of attack, the Canberra Raiders (25%) and the Brisbane Broncos (24%) have the highest attacking home advantage, while the Melbourne Storm (34%) and the Newcastle Knights (34%) have the highest percentage home advantage in terms of points conceded at home. While there can be a number of ways of quantifying home advantage, the data in Table 1 clearly supports its existence. The next task is to determine the role in the continuance of home advantage played by unconscious bias from NRL referees. Moreover, it is likely that the “referee effect” in home advantage is not homogeneous but rather varies across referees. The extent of this variation is under-researched, but it would be expected that such variables as experience would be an important determinant. Yet, consideration of the data shows that even among elite professional referees, considerable differences in performance emerge. Table 2 examines the win rate of NRL teams under a group of experienced NRL referees. To allow for greater consistency, the professional referees used in this sample were required to have refereed each team at least 10 times. The data in Table 2 indicate a considerable degree of variation in win ratio per team when controlled by different referees and some notable deviations from the average win rate for each team.

**Table 2:** Winning percentages for NRL teams under different leading professional NRL referees.

**Team**	**Referee 1**	**Referee 2**	**Referee 3**	**Referee 4**	**Referee 5**	**Referee 6**	**Referee 7**	**Average win rate**	**Win rate**
Auckland Warriors	48.48%	55.00%	44.44%	43.59%	51.06%	40.00%	NA	47.67%	0.57
Brisbane Broncos	58.49%	65.75%	66.00%	50.00%	60.42%	45.24%	57.69%	61.27%	0.67
Canberra Raiders	39.29%	55.88%	53.66%	42.11%	58.54%	28.13%	54.17%	53.70%	0.55
Canterbury B-Bulldogs	58.70%	42.19%	63.46%	56.76%	53.49%	57.14%	75.86%	58.53%	0.68
Cronulla S-Sharks	36.84%	49.02%	35.42%	54.90%	42.86%	38.24%	40.63%	51.28%	0.50
Manly W-Sea Eagles	65.52%	51.43%	42.42%	46.34%	55.00%	67.50%	70.83%	59.47%	0.67
Melbourne Storm	62.50%	33.33%	55.81%	59.26%	72.97%	71.19%	NA	64.95%	0.71
Newcastle Knights	45.95%	43.08%	50.00%	29.73%	53.70%	52.78%	36.67%	48.22%	0.52
North Q-Cowboys	29.17%	37.50%	37.50%	42.86%	40.00%	47.50%	NA	42.63%	0.39
Parramatta Eels	47.83%	51.92%	38.00%	40.00%	46.34%	48.78%	54.55%	49.03%	0.55
Penrith Panthers	36.84%	40.00%	63.64%	44.12%	50.00%	50.00%	32.00%	45.40%	0.53
South Sydney Rabbitohs	34.62%	27.27%	41.18%	55.81%	41.18%	28.57%	61.11%	42.37%	0.48
St George Ill-Dragons	47.92%	45.00%	46.67%	47.92%	58.49%	58.14%	52.94%	53.73%	0.60
Sydney Roosters	59.09%	62.22%	42.86%	67.35%	37.50%	57.89%	37.50%	53.73%	0.61
Wests Tigers	42.55%	37.93%	50.00%	40.48%	45.45%	40.43%	63.40%	46.89%	0.53

A number of points arise from consideration of Table 2. The Melbourne Storm win, on average almost 65% of their games, but even after a ten-game average, their winning percentages under our sample of experienced referees range from 33 to 73%. The Warriors win 55% of the time under Referee 2 compared to their average win of 47.67% under all referees; Brisbane does better under Referee 2 and Referee 3 and the Tigers fare significantly better under Referee 7 (63%) compared to their average winning ratio of 47%. None of this, of course, necessarily reflects unconscious bias but it is an indication that even among the most experienced senior NRL referees, their interpretations of rules and reaction to the playing styles of various teams differs. This potentially impacts on team performances and even results. It is in these areas that unconscious bias is likely to surface.

## Section 3—Decomposing the Referee Effect from Other Factors

The variations in outcomes by referees shown in Table 2 are strong indicators of inconsistencies in refereeing, even at the elite or highly experienced professional level but not necessarily as a result of unconscious bias. For this to be established we would need to show that match outcomes, particularly relating to the home team, are impacted by consistent, albeit unconscious decisions by the referee. There seems little doubt that refereeing decisions can impact the outcomes in NRL matches but, how important are they in aggregate and are they the result of unconscious bias? One way of moving toward this conclusion would be to quantify the extent to which referee decisions do make a difference to match statistics.

To examine this issue, points difference in a home game is used as a dependent variable and regressed in a random effects regression format against a number of variables thought to influence points differentials in favor of a home team, including decisions of the referee in terms of awarding penalties and ordering scrums^2^. The regression took the format:


(3) $$\begin{array}{l} y_{3jt}=\;\beta_{0j}+\;\beta_{1j}x_{6t}+\beta_{2j}x_{7t}+e_{jt} \end{array}$$

Where *y*_3*jt*_ = scrum difference and penalty difference (separate equations) represent the difference in scrums awarded to home and away teams across all teams and between the years 1978–2019 and act as the dependent variable. The independent variables, across the panel 1978–2019 are:

- Penalty difference, the difference in penalties awarded to the home team minus the penalties awarded to the away team / Scrum difference, the difference in home team fed scrums^3^.
- Crowd, represents the size of the crowd at the particular game.
- Crowd density, represents the ratio between maximum capacity and actual attendance at the particular game.
- A set of control variables which adjust for the quality of the team; EHGF (expected home points for), EHGA (expected home points against), EAGF (expected away points for) and EAGA (expected away points against)^4^.

We expect, given the influence of the control variables, that the direct indicators of referee input (and impact) would come through scrum and penalty differences, particularly penalty differences. The control variables chosen, EHGF and EAGA, do pick up variations in opposition team quality, albeit imperfectly. However, the value of using these variables is that they enable direct comparison with results obtained in earlier studies of referee impact such as Burnett et al. (2017). With data being drawn between the years 1978 to 2019, the impact of the two-referee system was also considered. For example, it would be possible to test for differences between the one and two referee systems by using slope dummies and a Chow test but even if this proved to be significant, it would impact on the intercept of the estimating equation rather than the slope of coefficient values on which our results are based. While it is also possible that referee appointments might be influenced by team match-ups or the quality of different games, we feel that the crowd density variable picks up some of these “big game” appointment effects. We did not specifically consider the distinction between day and night games as given the database, working with this variable would be difficult to accurately calculate. The results are shown in Table 3.

**Table 3:** Random effects regression with points difference as the dependent variable.

. xtreg Pointdifference Scrum difference Penalty Difference Crowd Crowd density EHGF EHGA EAGF EAGA,re						
Random-effects GLS regression			Number of obs = 6,185			
Group variable: Year			Number of groups = 40			
R-sq:			Obs per group:			
Within = 0.0327			Min = 55			
Between = 0.2811			Avg = 154.6			
overall = 0.0340			Max = 235			
			Wald $\chi_{\left(8\right)}^{2}$ = 217.15			
Corr(u_i, X) = 0 (assumed)			Prob > χ^**2**^ = 0.0000			
**Point difference**	**Coef**.	**Std. Err**.	**z**	**P>[z]**	**[95% Conf. Interval]**	
Scrum difference	0.1843591	0.0595091	3.10	0.002	0.0677234	0.3009948
Penalty difference	0.4658309	0.0613376	7.59	0.000	0.3456114	0.5860504
Crowd	0.0001119	0.0000334	3.35	0.001	0.0000464	0.0001773
Crowd density	−8.859138	0.9695093	−9.14	0.000	−10.75934	−6.958935
EHGF	0.2941757	0.0506288	5.81	0.000	0.1949451	0.3934062
EHGA	−0.1378962	0.0611651	−2.25	0.024	−0.2577776	−0.0180149
EAGF	−0.4162389	0.0616072	−6.76	0.000	−0.5369868	−0.295491
EAGA	0.1658631	0.053438	3.10	0.002	0.0611266	0.2705997
_cons	6.332417	1.436661	4.41	0.000	3.516613	9.148221
Sigma_u	0					
Sigma_e	16.876009					
rho	0 (fraction of variance due to u_i)					

In Table 3, all the chosen variables are significant. The coefficient on Scrum difference indicates that for every scrum win advantage, the home team adds an extra 0.2 of a point while for every additional penalty advantage they receive boosts the expected points difference by almost 0.5 of a point. Yet, in many ways, rugby league scrums are automatic and result from errors in play and are therefore quasi-independent from individual referee decisions. Similarly, some penalties are obvious, but many are not, as they are the result of referee interpretation^5^. It is in this area that unconscious bias is likely to emerge. Crowd size does not appear to be an important factor in home team points difference, but crowd density does, reducing home advantage by almost 8 points. The reasons for this are varied. One explanation is that a near capacity crowd (i.e., high crowd density) indicates an important match or show piece game and referees are likely to be attempting to stay neutral and more conscious of their performance. Another contributing factor is that in a show piece game the teams are likely to be more evenly matched, thereby reducing the scope for home advantage.

The results in Table 3 show that at least part of the home advantage (home team points difference) results from referee decisions. A related question is whether this referee contribution to home advantage diminishes with the experience of the referee. This is examined in Table 4. In this test, the referee list was initially divided into 5 tiers ranging from tier 1 (referees who have refereed over 300 games) to tier 5 (referees with <50 games). However, using tier 1 referees as the default, we were unable to find any significant differences in their impact on points difference. This seemed surprising, given it would appear likely that experience levels would be a significant point of departure in referee performance. This led to an aggregation of the referee tier dummies. In Table 3 we compare tier 1 referees against the rest (an amalgamation of tiers 2–5) and observe significant differences.

**Table 4:** Random effects regression—tier 1 referees against an amalgamation of tiers 2–5.

. xtreg Pointdifference Scrumdifference PenaltyDifference Crowd Crowddensity EHGF EHGA EAGF EAGA tierother,re						
Random-effects GLS regression			Number of obs = 4,758			
Group variable: Year			Number of groups = 31			
R-sq:			Obs per group:			
Within = 0.0279			min = 55			
Between = 0.3558			avg = 153.5			
overall = 0.0299			max = 235			
			Wald $\chi_{\left(9\right)}^{2}$ = 146.52			
Corr(u_i, X) = 0 (assumed)			Prob > χ^**2**^ = 0.0000			
**Point difference**	**Coef**.	**Std. Err**.	**z**	**P>[z]**	**[95% Conf. Interval]**	
Scrum difference	0.1610133	0.0660516	2.44	0.015	0.0315546	0.2904721
Penalty Difference	0.3016788	0.0669968	4.50	0.000	0.1743675	0.4329902
Crowd	0.0000443	0.0000419	1.06	0.290	−0.0000378	0.0001265
Crowd density	−7.225506	1.146926	−6.30	0.000	−9.47344	−4.977571
EHGF	0.3358963	0.0563924	5.96	0.000	0.2253693	0.4464233
EHGA	−0.1604833	0.0693742	−2.31	0.021	−0.2964542	−0.0245123
EAGF	−0.4856779	0.0711955	−6.82	0.000	−0.6252185	−0.3461373
EAGA	0.180652	0.0591345	3.05	0.002	0.0647505	0.2965536
Tierother	−1.535265	0.6800083	−2.26	0.024	−2.868056	−0.202473
_cons	7.777761	1.765354	4.41	0.000	4.317731	11.23779.
Sigma_u	0					
Sigma_e	16.953868					
Rho	0 (fraction of variance due to u_i)					

As with Table 3, all variables are significant, except the crowd numbers. Most importantly for our analysis, the tier other dummy is significant and suggests that the home advantage points difference is increased under less experienced referees when compared to the tier 1 referees. In other words, the other factors that contribute to home advantage become more important under less experienced referees. This may indicate the less experienced referees are less successful in dealing with home crowd noise and other factors that potentially influence referee decisions.

## Discussion

This paper found that in the National Rugby League, when observed over a number of trials, clubs fare significantly better or worse under particular referees. First, we demonstrated that clubs consistently have different outcomes in terms of result and points scored under different referees, even when those referees are the most experienced senior referees in their field. Second, it has shown that referees influence points scored in home games (and therefore home advantage) through their decisions over penalties and scrums. Lastly, it has been shown that home advantage is increased under less experienced referees. Taken together, the results show that the search for consistent and objective rulings in refereeing decisions is still some way off. We also believe that these inconsistencies represent unconscious bias on the part of referees in the way they interpret and police particular aspects of the game and their reaction to team styles, particular players, and unfolding on-field events.

Controlling professional sporting events, however, is not an easy task and referees will make mistakes. In an age of increasing electronic surveillance these mistakes are highlighted and as a result, extensive efforts are made by sport organizations to educate and train referees to provide consistent and objective rulings. However, biases can be hidden in very complex and strategic ways, and referees, even at the experienced level, are rational agents who can learn how their organization functions and adjust accordingly, which makes detection difficult (Agarwal, 2020; Osório, 2020). Thus, while NRL referees might have honorable intentions, the reality is that their judgement behaviors are the subliminal product of both the situation or context (e.g., organizational review processes, specialist training, positioning, crowd effect, stadium, emergent player actions) and the person themselves (e.g., rule knowledge, individual motivations, background, memory structures, recollections). Moreover, under the revised one-referee system, when NRL referees are regularly at the heart of stormy discussions which provide a substantial source of material for both media and fans (Dohmen and Sauermann, 2016), determining how and why some referees still stress some aspects of information to the neglect others would seem an even more important factor for organizations to try and resolve.

It will be a challenge for sporting bodies to reconcile educational processes with how referees subconsciously overweight some aspects of information to the neglect of others. We would further argue that a re-conceptualization of the expert sport referee (i.e., consistent performer who displays minimal mistakes in judgment) requires a potential paradigmatic shift from the expert referee as product, to the evolution of referee expertise as always becoming, continually learning, a never-ending journey of ongoing professional discovery (Turner et al., 2012; O'Brien and Rynne, 2020). Organizations that continue to be preoccupied with problems and deficit run the risk of missing data related to how learned stereotypes that are automatic, unintentional, and deeply ingrained influence the way in which sport referees react and behave in the presence of crowds and crowd noise. In this regard, perhaps the solution is how empirical and experiential knowledge can be better brought to life or transformed into learning experiences that authentically address the development and performance preparation of professional referees. This transcending the individual as a decontextualized ‘unit of data analysis’, we believe, reinforces the underlying messiness of performer-environment systems and the growing idea that different approaches to developing referees in their role are required.

## Data Availability Statement

The data analyzed in this study is subject to the following licenses/restrictions: Dataset belongs to University of Queensland. Requests to access these datasets should be directed to katherine.obrien@qut.edu.au.

## Author Contributions

KO'B contributed the abstract, introduction, Section 1—Unconscious bias in sport refereeing, and discussion. JM contributed Section 2—the empirics of unconscious bias in rugby league and Section 3—decomposing the referee effect from other factors. All authors contributed to the article and approved the submitted version.

## Funding

This work was supported by QUT Women in Research Grant.

## Conflict of Interest

The authors declare that the research was conducted in the absence of any commercial or financial relationships that could be construed as a potential conflict of interest.

## Publisher's Note

All claims expressed in this article are solely those of the authors and do not necessarily represent those of their affiliated organizations, or those of the publisher, the editors and the reviewers. Any product that may be evaluated in this article, or claim that may be made by its manufacturer, is not guaranteed or endorsed by the publisher.

## Footnotes

^1^On that basis, another way of quantifying home advantage would be to examine the shift in the probability of winning at home and away.

^2^In rugby league, scrums have taken a quasi-automatic nature with teams awarded the scrum (that is getting to put the ball in) almost always winning the scrum.

^3^Interchangeable for the dependent variable.

^4^These specific control variables take the form recommended by Boyko et al. (2007).

^5^In general, rugby league penalties are the sole responsibility of the referee and not subject to intervention by the video referee. This has changed slightly with the introduction of the “captains challenge” whereby teams may dispute some referee decisions and ask for them to be referred to the video referee.
